# 100 years after Griffith: From brittle bulk fracture to failure in 2D materials

**DOI:** 10.1557/s43577-022-00379-2

**Published:** 2022-08-24

**Authors:** Daniel Kiener, Seung Min Han

**Affiliations:** 1 Department of Materials Science, Montanuniversität Leoben, Leoben, Austria; 2 Department of Materials Science and Engineering, Korea Advanced Institute of Science and Technology, Daejon, Republic of Korea

## Abstract

Brittle fracture and ductile failure are critical events for any structural or functional component, as it marks the end of lifetime and potential hazard to human life. As such, materials scientists continuously strive to better understand and subsequently avoid these events in modern materials. A century after the seminal initial contribution by Griffith, fracture mechanics has come a long way and is still experiencing vivid progress. Building on classical fracture testing standards, advanced *in situ* fracture experiments allow local quantitative probing of fracture processes on different length scales, while microscopic analysis grants access to chemical and structural information along fracture paths in previously unseen detail. This article will provide an overview of how these modern developments enhance our understanding of local fracture processes and highlight future trends toward designing strong yet ductile and damage-tolerant materials.

**Graphical abstract:**

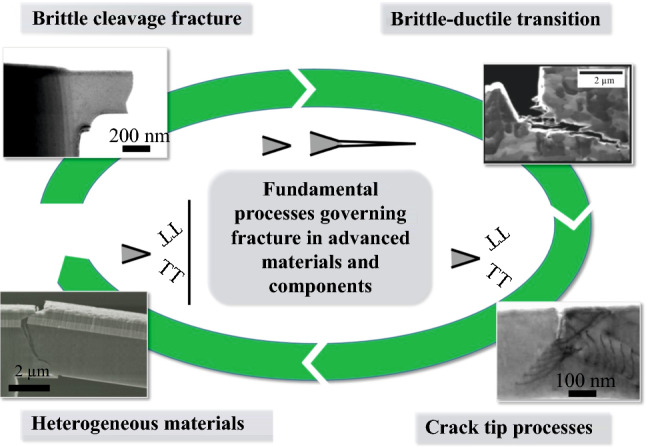

## A historical perspective

At the beginning of the last century, scientists had uncovered several fundamental aspects of materials. For example, they realized that most materials, such as common metals and ceramics, possess a crystalline structure and that their elastic properties are related to the bonding strength among atoms. However, there remained a significant deviation between the estimated theoretical strengths of materials and experimental observations. In fact, among others A.A. Griffith (June 13, 1893–October 13, 1963) noticed that the fracture strength of glass was in the range of 100 MPa, several orders of magnitude below the theoretical prediction of *E*/10, with *E* being the elastic modulus resultant of the atomic binding energies, which would give rise to values in the range of 10 GPa.

Griffith^1^ ascribed this behavior to invisible defects in the glass and experimentally verified this hypothesis by introducing target artificial defects, demonstrating a roughly constant product between fracture stress and square root of the defect size. Deriving a theory for brittle fracture of solids proved challenging due to the mathematical treatment of the stress singularity at the crack tip. Thus, Griffith analyzed the change in free energy for a loaded plate containing a crack upon crack extension in a linear elastic setting, the difference relating to the relaxation of strain energy within the material close to the crack and the increase in surface energy due to the crack presence.

While the model well-described data for brittle ceramics, such as glass, it was largely ignored in the engineering community, as the observed values in energy release necessary for crack propagation determined for engineering metals exceeded those predicted by the model of Griffith by orders of magnitude. Evidently, something else had to happen during ductile fracture of metals. In fact, they plastically deform well below their theoretical strength, facilitated by the generation and movement of dislocations, as postulated in 1934 independently by Taylor,^2^ Orowan,^3^ and Polanyi.^4^ Notably, while Orowan and Polanyi published their works back to back in the September issue of *Zeitschrift für Physik*, Taylor preceded them by roughly three months with his work in the July issue of the *Philosophical Transactions of the Royal Society of London*.

The energy dissipated by plastic deformation was the reason the Griffith theory did not apply to metals, and it took until the late 1940s for Irwin^5^ and Orowan^6^ to include this plastic dissipation to the total energy release, upon which they were able to successfully describe fracture of metals. Irwin furthermore derived the resultant strain energy release rate *G*, describing the rate at which energy is absorbed by a growing crack,^7^ reformulated the stress singularity problem at the crack tip, and introduced the stress intensity factor (SIF),^8^ thereby laying the foundation of today’s linear elastic fracture mechanics (LEFM) concepts.

To account for significant plastic deformation in front of the crack, where LEFM would not be valid anymore, the crack tip opening displacement (CTOD) was later suggested by Wells^9^ as a critical parameter. In 1968, Rice^10^ generalized the energy release rate to nonlinear material behavior, resulting in the J-integral as a parameter describing elastic–plastic failure, and Hutchinson demonstrated in the same year that this J-integral can be utilized to describe the stress state at a crack tip for an elastic–plastic material.^11^ Finally, Shih^12^ linked these two parameters describing fracture in the framework of elastic–plastic fracture mechanics (EPFM). Together, these works build the foundation of standards developed for fracture toughness, fatigue crack propagation, environment-assisted crack propagation, as well as for damage-tolerant design of components.^13^

**Figure 1:**
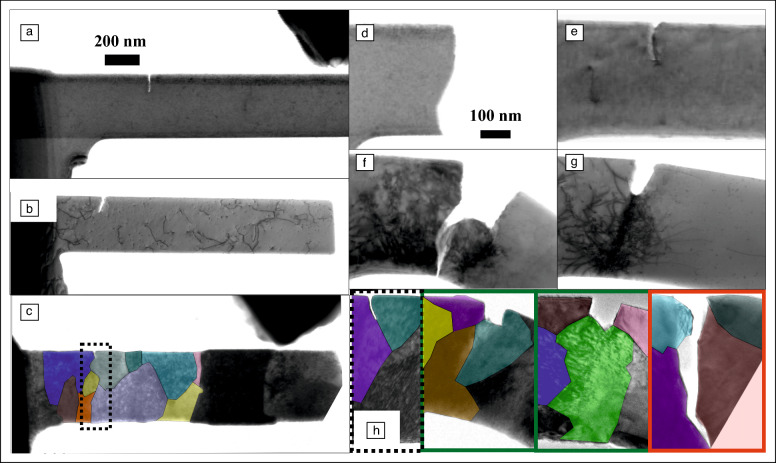
*In situ* transmission electron microscope images of cracks situated in (a) a dislocation free, (b) a dislocation containing crystal, or (c) at an interface. Crack tip processes can comprise (d) brittle cleavage or (e) dislocation emission, causing either (f) ductile fracture or (g) plastic blunting. (h) Interface cracks either support dislocation nucleation and crack tip blunting or cause intercrystalline fracture.

In a sense, the mathematical frameworks relate to the fracture behaviors encountered for different materials and microstructures, which could contain movable dislocations and various interfaces and other microstructural components or be devoid thereof (**Figure ****1**a–c). Focusing on crack tip processes in metals as their most prominent toughening mechanism, depending on the crystal structure and loading situation, the material can fail via brittle cleavage, emit some shielding dislocations and eventually fail, or plastically blunt the crack tip (Figure 1d–g). Regarding situations containing interfaces, those can either promote dislocation nucleation for crack tip blunting or cause intercrystalline failure, as exemplarily shown in Figure 1h.

## Current cutting edge fracture assessment in advanced materials

### Fundamental considerations of fracture processes

Current trends in improving the strength of materials in order to reduce material and energy consumption constantly push the refinement of microstructures into the ultrafine-grained (ufg) and nanocrystalline (nc) regime. Concurrently, thin films and multilayer structures with micrometer- to nanometer-layer thicknesses and related microstructures are being developed as structural or functional components. While the nanoscale microstructure dramatically benefits the material strength,^14,15^ it can severely limit the achievable ductility,^16^ and thereby also the plastic energy dissipation in front to the crack tip. Therefore, high strength and toughness represent an almost exclusive combination in conventional engineering materials,^17^ and even more so for nanostructured materials^18^ or thin films.^19^ With shrinking grain sizes to the micron- and submicron regime, dislocation slip distances accordingly reduce, which concurrently limits the amount of energy to be dissipated. For microstructures in the deep submicron to nanometer regime, deformation is generally interface-mediated, and failure mechanisms commonly change from a ductile transcrystalline characteristic in the coarse-grained variant to a rather brittle intercrystalline fashion for the nanostructured material modification. Furthermore, smaller grain sizes cause rather flat fracture surfaces, a clear indication that extrinsic toughening contributions such as crack deflection and grain bridging are also reduced.^20^

**Figure 2:**
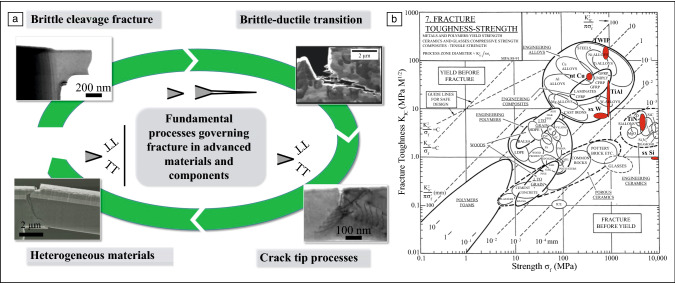
(a) Schematic of different fracture processes that govern the failure behavior of modern materials and structures. (b) Ashby map showing fracture toughness as a function of material strength, where dashed lines indicate estimate radius of the plastic zone and crack tip opening displacement in millimeters, respectively. Some of the materials covered in this issue are indicated for reference. Modified from M.F. Ashby, *Materials Selection in Mechanical Design*, 2nd ed. (1999), with permission from Butterworth-Heinemann.

Before diving deeper into the current challenges and progress made in understanding fracture and failure mechanisms, as schematically depicted in **Figure ****2**a, it might be instructive to take a closer look at typical dimensions, time scales, and local strain state involved, as this will also set thresholds for potential additional insights. Evidently, for brittle fracture the initial bond breaking events occur at the crack tip within lattice spacing, thus in the range of 0.2–0.5 nm, in an area strained to roughly 10% elastic strain. Afterward, we can for simplicity assume that the crack instantaneously propagates at approximately the transverse acoustic wave velocity of the material,^21^ which ranges in values of 4000–6000 m/s for common metals, such as Mg, Al, Ti, Fe, Cu, or W. Relating this to microscopic imaging, evidently the crack will surpass any micron-sized field of view in fractions of microseconds, posing severe challenges to any imaging-based investigation technique.

For ductile failure involving the emission of dislocations from a crack tip to blunt it, the situation is a bit less dramatic, but still challenging. In fact, we can initially assume an elastically strained region involving elastic strains of 0.2–2% surrounding the crack tip of interest, thereby setting the region of interest to span distances from tens of nanometers to many micrometers depending on the strength of the concerned material. In this region, assuming the Orowan equation^22^ suited for an approximate assessment, dislocations will nucleate and/or propagate depending on the stress acting on them, which is given by the interplay between driving forces from the crack tip stress field on the one hand and the lattice friction of the material under investigation and potential back stresses of previously emitted dislocations on the other hand. For low Peierls barrier materials, such as Cu, and realistic dislocation densities, dislocation velocities are still in the range of several hundred m/s,^23^ sliding over large travel distances. For high Peierls barrier metals such as W at low homologous temperatures and with sufficient distance to the crack tip, this approaches the applied far-field conditions, with experimentally still challenging but potentially accessible slip velocities of several µm/s.^24^

Referring to the Ashby map in Figure 2b, it is instructive to regard the estimated process zones, radius of the plastic zone and crack tip opening displacement, respectively, indicated by dashed lines, as this relates to the area of interest for *in situ* observation. Furthermore, some of the materials governed by related in-depth articles on this topic in this issue of *MRS Bulletin*^36,47,50,51,65,79,81^ are indicated to provide the interested reader with more details for a wide range of materials.

### Influence of interface chemistry on intercrystalline fracture

As previously noted, severe grain-size reduction to submicrometer or even nanometer length scales causes a transition from transcrystalline to intercrystalline failure. Consequently, it becomes important to strengthen the interfaces, as they pose the weakest link in the microstructure. In the concept of grain-boundary complexion engineering,^25^ this can be attempted on different interface length scales, for example, using ductile phases in a nanocomposite approach,^26,27^ by extent amorphous interphases,^28^ or local interface segregation.^29,30^ While all of them hold promise and have been demonstrated to result in significant improvements of material toughness, the latter seems the most elegant approach with respect to general and scalable applicability, sustainable use, and recyclability of materials, as only minor fractions of alloying or rather doping elements are necessary to segregate or decorate the grain boundaries. Selection of the respective grain-boundary doping elements should be guided on the one hand by *ab initio* calculations, looking at the effect of the respective elements on strengthening or weakening interface cohesion,^31^ and on the other hand by thermodynamic considerations of the segregation tendency of the employed dopant,^32^ as the strength of segregation defines the efficiency of dopant use in modifying the interface properties.

A detailed understanding of the fundamental structural transitions responsible for increased interface cohesion and potentially a simultaneous reduction in dislocation emission stress intensity of such interfaces,^33^ requires primarily correlation to the local chemistry and in a later stage also to local mechanical properties. The fact that mere doping with grain-boundary modification elements is considered poses a significant challenge to material characterization, on the one hand to potential structural changes at the interface,^34^ and on the other hand to the chemical analysis of interface excess segregation.^35^ The methods of choice that natively come to mind in addressing those are transmission electron microscopy (TEM) and atom probe tomography (APT), respectively. As reviewed by Dehm and Cairney,^36^ recent developments pushing these highly advanced techniques even further are ongoing in order to enable a better understanding of the influence of interface chemistry and structure on fracture processes.

### Determining stress state and dislocation processes near interfaces

Once the structural and chemical configuration at the interfaces are resolved, the next question refers to the respective effects on local cohesion, decohesion stresses, as well as dislocation nucleation and propagation events at or in close proximity to the respective interfaces. These processes typically occur within the material, because the stress state is of importance for the failure mechanism. Studying the material at the dislocation level again points to TEM techniques. But while they proved themselves very powerful for studying fracture processes from the early days until today, covering everything from bulk materials to two-dimensional structures,^37,38^ this also comes at the requirement for electron transparency of the thin specimen. As such, investigating a plane strain condition at the crack tip is possible only for rather strong and brittle materials, such as Si,^39^ while for most semi-brittle-to-ductile materials only plane stress-dominated conditions can be examined in TEM.

To study bulk fracture processes *in situ* in a confined situation as demanded in fracture standards, other means to internally address the materials are required. Bulk-like fracture experiments can, for example, be conducted within scanning electron microscopes (SEMs). This probes a bulk failure condition, but offers only to access the emerging surface features and related surface strain fields.^40^ However, recent advances in electron channeling contrast imaging (ECCI) permit not only visualization of surface features, but also dislocation structures in the near subsurface material.^41^ These structures emerging upon fracture and failure of bulk materials can be enhanced by surface strain fields and local dislocation density analysis from electron backscatter diffraction (EBSD) to derive a comprehensive picture of the failure process.^42,43^ Importantly, such a combination of techniques examining bulk samples can resolve individual dislocations and provide high strain resolution over large areas. To date, they still suffer from temporal resolution, because tilting for optimum conditions and scanning for data acquisition limit the temporal resolution. Even though significant progress toward more precise and faster data collection is made, for example, by direct electron detectors in conjunction with smart acquisition schemes,^44^ the experiments are still considered as quasi-stationary, and stiff intrinsically displacement-controlled machines are required for a well-controlled experiment.

Alternatively, a fracture experiment can be monitored in a synchrotron. Recent advances in focusing optics providing high brilliance nanobeams in combination with much faster two-dimensional detectors enable access to the local strain field in front of the crack.^45^ Typically, the method lacks the sensitivity to assess individual interfaces in a nanostructured material, but probes the average material responses within the illuminated volume. Nonetheless, the amount of information gained involving phase, crystallography, dislocation density, microstrain, etc., is impressive. Even more so, as beam diameters well below 100 nm are considered almost a standard feature. In analogy, by employing diffraction mapping techniques in the TEM^46^ similar information content as for synchrotron nanobeam diffraction experiments can be acquired, but with nanometer resolution, and complemented by additional benefits offered by TEM techniques, such as visualization of individual crystal defects. Furthermore, using diffraction techniques, thicker samples can be investigated, thereby reducing the effect of nearby free surfaces, while radiation hard direct electron detectors again serve to dramatically increase the data acquisition rate. More details on the exciting experimental developments toward analyzing the strain state and microstructural modifications during fracture processes are covered by Gammer and An.^47^

### Fracture processes in complex engineering alloys

Overcoming the strength–ductility paradigm and empowering strong materials with sufficient toughening for a safe design are key challenges embarked in further pushing the boundaries of complex engineering alloys. Due to the multitude of inflicting and intertwined aspects of microstructural design possibilities in conjunction with restraints on, for example, lightweight design and high-temperature creep strength, and at the same time ensuring failure tolerance, corrosion resistance, and reusability, high-performance engineering materials such as titanium aluminides (TiAl)^48^ or Ni-based superalloys^49^ have been iteratively improved with respect to synthesis and processing techniques involving significant experimental and theoretical efforts. Similar challenges apply to emerging materials, such as bulk metallic glasses, where multiple factors such as chemistry, processing, size, and temperature affect the brittle-to-ductile transition among other properties, as addressed by Ryu et al.^50^

When aiming to improve fracture properties in such complex microstructures, while the experimental standards and evaluation schemes are established, it still remains challenging to assign the magnitude of toughening contributed by a certain microstructural feature to the overall fracture toughness or R-curve behavior. As detailed on the example of different model microstructures of TiAl alloys by Pippan and Hohenwarter,^51^ even within the validity of LEFM, it remains a demanding task to assign or differentiate contributions arising from, for example, crack deflection, crack tip bridging and wedging, and ligament shear to the overall fracture toughness deduced from testing the whole bulk structure. While some insights are possible based on postmortem analysis of the fracture surface and *in situ* testing of bulk-like components as regarded in the previous paragraph, there are also limits to the local information gain. In this regard, small-scale testing provides the opportunity to specify the volume to be examined,^52^ enabling to assess individual microstructural features^53^ or interfaces.^54^ This would allow, for example, to identify the weakest link in the structure for a targeted improvement.

### Locally specific fracture mechanical examinations

Miniaturized mechanical testing can deliver local and specific information of material characteristics, being it strength or fracture toughness.^55^ While small-scale experiments have matured over the last years, contributing to a rather complete understanding of plasticity in confined volumes,^56^ the situation is naturally somewhat more complicated concerning miniaturized fracture experiments,^57^ where some limitations arise. This is most and for all linked to length-scale requirements for conducting valid fracture tests.^58^ In fact, even for reasonably strong low toughness materials, implying fracture toughness values in the single digit MPa m^0.5^ range, the plastic zone can reach micrometer dimensions. This can be substantial for small-scale samples, rather than diminishing as required by Irwin’s work.^8^ Furthermore, at small enough scales and high stresses, materials that commonly fracture in a brittle nature can deform plastically. Although this enables studying their deformation mechanisms,^59^ it might severely impact the resultant fracture observations. Furthermore, while for brittle materials valid and geometry-independent fracture toughness values can be deduced given a sufficiently sharp pre-crack,^60^ for semi-brittle or ductile materials small-scale fracture experiments might only measure size-affected properties.^58,61^ In analogy to macroscopic fracture testing, this can be mitigated to some extent by larger specimens or progressing from LEFM to EPFM analysis,^62^ but even there certain limits prevail. Eventually, if not enough energy is stored in the system to fracture it, it will just plastically tear.

Nonetheless, miniaturized testing techniques open many possibilities to not only study local strength but also fracture properties. In fact, even miniaturized designs for different fracture modalities have been suggested,^63^ enabling to study mixed mode fracture on a microstructural length scale. Furthermore, also local fatigue processes can be examined,^64^ thereby addressing aspects spanning from the confined single-crystal response via the impact of individual interfaces to the bulk behavior of complex structures. On the one hand, such insights are detrimental for a fundamental understanding and improvement of fracture and fatigue processes of advanced bulk, layered, topological, or hierarchical materials. On the other hand, the ongoing miniaturization trend demands for examining size-affected fracture characteristics of miniaturized objects employed in everyone's daily life, for example, in micro-electromechanical sensing and actuation applications in cars and cell phones. Further details addressing this topic are provided by Jaya.^65^

### Fracture processes in nanolayered composites

**Figure 3:**
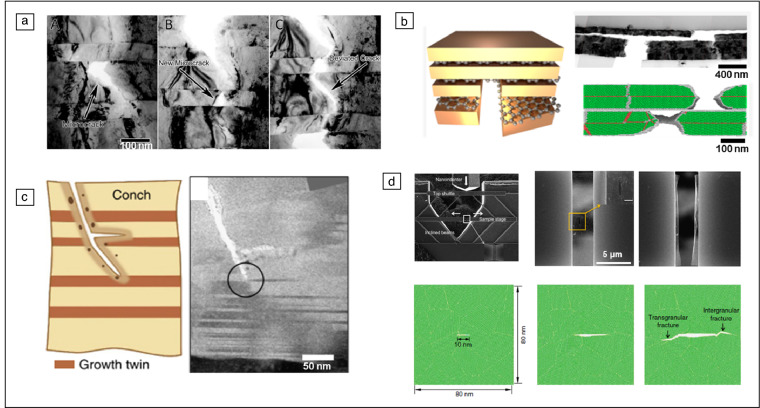
Fracture processes in nanolayered and 2D materials: microcracks deviated (a) at Cu/Nb interfaces^72^ or (b) deflected at Cu/graphene interfaces.^75^ (c) Schematic and transmission electron miscrocope image of an aragonite conch shell, with the crack tip arrested by nanoscale twins.^76^ (d) Fracture toughness testing of pre-cracked freestanding graphene using a push-to-pull device and a complementary molecular dynamics simulation showing brittle crack propagation from the initial defect.^80^

Nanotwinned metals and nanolayered composites are engineered materials in which a high density of interfaces is present, and the interfacial characteristics play an important role in determining the mechanical properties including fracture (**Figure ****3**). Twin boundaries are of low energy and high symmetry, while interfaces in nanolayered composites are of higher energy and disorder, and can thus act as dislocation obstacles as well as allowing to store some dislocations at the interfaces. As a result, interesting fracture responses are observed in nanotwinned and nanolayered composites. *In situ* TEM tensile testing showed that ultrafine-grained Cu with nanoscale twins possesses an interesting toughening mechanism, where the generated cracks were arrested by the twin boundaries and the twins served as crack-bridging ligaments.^66^ Other studies revealed a length scale-dependent brittle-to-ductile transition in nanotwinned metals, where cleavage along the twin boundary^67^ and ductile failure due to dislocation plasticity above and below a critical spacing, respectively, were reported.^68^

Nanolayered composites also contain a high density of coherent and incoherent interfaces that can hinder dislocation as well as crack propagation. Incoherent interfaces were reported to have the ability to absorb dislocations, which can extend the lifetime of nanolayered composites^69–71^ and hinder or block crack propagation^72,73^ (Figure 3a). In a Cu-graphene-nanolayered composite shown in Figure 3b, graphene with high in-plane strength and stiffness was able to arrest cracks, hence enhancing the robustness against fatigue-induced fracture.^74,75^ Furthermore, fracture in biological nanocomposites such as nacre,^76^ oyster shells,^77^ or biological exoskeletons^78^ received much interest due to the naturally occurring high fracture toughness. For example, nacre consists of a hierarchical structured composite containing nanotwins that provide resistance against crack propagation (Figure 3c), thereby resulting in high toughness.^76^ Nanolayered composites, whether an engineered or a naturally found material, therefore possess unique fracture properties that are governed by the existing high density of interfaces, as further detailed in a review by Zhao et al.^79^

### Fracture mechanics at atomic dimensions in 2D materials

As reviewed in detail by Steinbach et al.,^81^ 2D materials such as graphene or boron nitride are recently being used in a variety of engineering applications, and several studies aiming to understand deformation and fracture in 2D materials have been reported. In 2D materials, fracture is mostly initiated at defects, such as cracks, vacancies, dislocations, and grain boundaries.^82^ Testing of single or a few-atomic-layer-thick 2D materials poses a great experimental challenge, but has progressed significantly over the years. In an early experimental study, mechanical properties of graphene were probed using atomic force microscopy, where graphene flakes placed on arrays of circular holes were indented up to the point of rupture.^83^ In a more recent study, fracture toughnesses of graphene^80^ as well as MoSe_2_^84^ were measured using *in situ* tensile testing by a push-to-pull device (Figure 3d). An initial pre-crack was placed on the prepared graphene film using FIB. Tests have been conducted with varying initial crack sizes and corresponding fracture stresses were measured, which closely follow the Griffith theory despite being only a few atomic layers in thickness.^80,85^ The well-known Griffith equation states that the stress required for crack propagation is inversely related to the square root of the initial defect size.^1,13^ Thus, controlling the size and frequency of flaws in such 2D material is crucial for designing 2D material composites with sufficiently high toughness. Because some defects in large-scale material systems are inevitable, a number of studies were directed toward utilizing intentionally placed patterns for achieving better deformability.^86,87^ Thus, 2D materials with atomic layer thickness still follow what we learned from larger length-scale materials, as controlling the number and size of defects leads to different fracture strengths, according to the Griffith criterion proposed back in 1921.

## Future trends and needs to unravel fracture processes

Significant progress has been made and there is a wide suite of established, novel and emerging experimental techniques to study the local and abrupt nature of failure and fracture processes on a fundamental level. The same holds true for advanced modeling techniques aiding our understanding of elemental fracture events in advanced structures (e.g., see Figures 2, 3). It would appear that a complementary experimental and computational study of fracture processes examining and bridging the different involved length scales would be a valuable effort. In principle, all required capabilities exist, and the overarching premise of such an endeavor could be to derive at comprehensive understanding of the individual contributions to the overall toughness of complex advanced materials or structures.

However, some challenges also remain along this trajectory. On the mechanical side those relate to the need for truly displacement-controlled testing devices in conjunction with smart data acquisition schemes to capture fast and rare events at high fidelity. In terms of visualization, there remains a need for ultra-fast imaging techniques if the community attempts to progress from quasistatic to dynamic *in situ* imaging of fracture processes. Finally, there is also demand for advanced fracture mechanical frameworks that account on the one hand for experimental limitations, such as confined volume or component requirements, and on the other hand for material complexities, such as crack propagation in inhomogeneous materials or topological/hierarchical structures.

To conclude, there is a vast and rich range of opportunities for young creative minds and experienced scientists to jointly advance the field. It is not certain whether this will occupy the community for the next century, but certainly for a good number of years to come.

## Data Availability

Data will be provided by the authors upon reasonable request.

## Code availability

Not applicable.
